# Progressive Lung Consolidation in HIV Amidst the COVID-19 Pandemic: Evaluating Probable Cytomegalovirus Pneumonia and the Importance of Early Empirical Treatment

**DOI:** 10.7759/cureus.65921

**Published:** 2024-08-01

**Authors:** Sargunann Naidu Krishnasamy Naido, Suria Mano Geran, Nashriq Khan Adam Khan, Noralfazita An

**Affiliations:** 1 Diabetes and Endocrinology, Whittington Hospital NHS Foundation Trust, London, GBR; 2 HIV and Infectious Diseases, Hospital Sultan Abdul Halim, Sungai Petani, MYS; 3 Stroke Medicine, Russells Hall Hospital, The Dudley Group NHS Foundation Trust, Dudley, GBR; 4 Cardiology, Queen's Hospital, Barking, Havering and Redbridge University Hospitals NHS Trust, Romford, GBR; 5 Acute Medicine, Norfolk and Norwich University Hospitals NHS Foundation Trust, Norwich, GBR

**Keywords:** non-resolving pneumonia, empirical antiviral therapy, cytomegalovirus pneumonia, hiv aids, covid-19

## Abstract

We present the case of a young male who was diagnosed with HIV in 2012. However, his initiation of antiretroviral therapy (ART) was delayed until 2018 due to issues related to his acceptance and acknowledgment of the disease. In April 2021, the patient presented with hemoptysis, shortness of breath, and constitutional symptoms. Initial extensive workup for tuberculosis (TB) and other respiratory pathogens returned negative. Despite this, he was treated for smear-negative pulmonary TB and pneumocystis pneumonia (PCP) and was subsequently discharged. He then had recurrent hospital admissions due to worsening respiratory symptoms, with short intervals between recovery and recurrence. Each admission saw an increase in his oxygen requirements. Throughout these hospitalizations, tests for coronavirus disease 2019 (COVID-19) were consistently negative. TB and PCP treatment continued throughout his admissions. Despite various treatments, his condition continued to deteriorate. A DNA polymerase chain reaction (DNA PCR) test for cytomegalovirus (CMV) was eventually conducted. Unfortunately, the patient succumbed to progressive respiratory failure, and the CMV DNA PCR returned positive a week after his death. In the era of COVID-19, this case underscores the importance of early diagnosis and timely antiviral treatment.

## Introduction

Cytomegalovirus (CMV), a member of the Betaherpesviridae subfamily, is a critical opportunistic pathogen in HIV patients. It exploits immune system evasion mechanisms, exacerbating HIV pathogenesis by altering lymphocyte function. While initial CMV infection often presents as a mild, self-limiting illness with symptoms like fever, fatigue, and lymphadenopathy, it becomes significant in AIDS, particularly with CD4+ counts below 50 cells/mm³ [1]. Accurate diagnosis of CMV pneumonia in advanced HIV requires a comprehensive assessment of clinical, radiological, microbiological, and cytopathological features. CMV viral load testing is essential for guiding preemptive therapy, diagnosing active disease, and monitoring treatment response. In a study by Zhao et al., up to 77% of CMV-positive patients had a CD4 count of less than 50 cells/µL, highlighting this threshold as a risk factor for HIV-CMV coinfection. Therefore, initiating antiretroviral therapy (ART) promptly in patients with HIV/AIDS is recommended to promote immune reconstitution and prevent opportunistic infections. Additionally, CMV-DNA screening in plasma and urine should be performed when the CD4+ T lymphocyte count is less than 200 cells/µL, particularly when it is below 50 cells/µL [2].

## Case presentation

We present a case of a 29-year-old male diagnosed with HIV in 2012. He contracted the virus via an unprotected heterosexual relationship and his CD4+ was 508 cells/microlitre (13%) in 2012. Despite his diagnosis, the initiation of first-line highly active antiretroviral therapy (HAART) was delayed until 2018 due to stigma and denial. The patient defaulted on treatment within a year, and by March 2021, his CD4+ counts had fallen to 107 cells/μL.

In April 2021, he presented with hemoptysis, anorexia, significant weight loss (20 kg over two months), night sweats, and intermittent fever for five months. Physical examination revealed four palpable sub-centimetre submandibular lymph nodes. Workup for *Mycobacterium Tuberculosis* (MTB) which included a Sputum Gene Xpert MTB/RIF and Sputum MTB culture and sensitivity after an eight-week incubation period revealed negative results. A chest X-ray (CXR) showed bilateral lung consolidation. Given his immunocompromised state, endemic TB, and CXR findings, he was treated empirically for smear-negative pulmonary TB and pneumocystis pneumonia (PCP). First-line anti-TB (oral Isoniazid, rifampicin, ethambutol, pyrazinamide, and pyridoxine) were initiated. He received a tapering dose of oral prednisolone, followed by a 21-day course of oral sulfamethoxazole and trimethoprim. He was discharged after five days.

**Figure 1:**
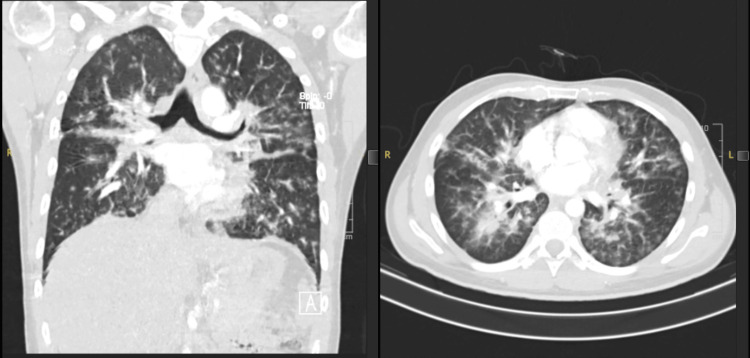
Coronal view (left) and axial view (right) of CTPA shown in the lung window CTPA done during the patient's second admission in April 2021 to exclude pulmonary embolism demonstrates multifocal, patchy, ground-glass opacities mixed with interstitial opacities seen at both lung fields, worst at the superior segment of the right lower lobe, left lingula segment, and perihilar region with atelectatic changes at both lung bases CTPA: computed tomography pulmonary angiogram

The patient returned 10 days later with worsening dyspnea and low-grade fever. He was diagnosed with hospital-acquired pneumonia (HAP) and treated with ceftriaxone for one week. The treatment for TB and PCP was continued. A CT pulmonary angiography (CTPA) showed no pulmonary embolism but revealed multifocal ground-glass and interstitial opacities, primarily in the right lower lobe, left lingula, and perihilar regions (Figure 1). Sputum cultures did not isolate any pathogen and blood cultures grew no organisms for five days. After over a week in the ward, he was discharged once his oxygen requirements decreased.

The patient was readmitted shortly after with dyspnea, pleuritic chest pain, and hemoptysis, and was again treated for HAP. A coronavirus disease 2019 (COVID-19) polymerase chain reaction (PCR) test was negative. Additionally, he reported worsening diarrhea over the past week. Stool samples were negative for ova, cysts, *Cryptosporidium spp*., *Microspora spp.*, *Cyclospora spp.*, *Isospora spp.,* and *Clostridium difficile*. Suspecting TB colitis, a contrast-enhanced CT abdomen, and colonoscopy were scheduled but not performed due to COVID-19 restrictions. He was treated with IV piperacillin-tazobactam and oral metronidazole. A possible TB colitis/CMV colitis diagnosis was made and therefore sent for an ophthalmologic screening for CMV retinitis, which was normal. He ultimately recovered and was discharged.

In mid-May, the patient presented with dyspnea, productive cough, and intermittent fever, requiring increasing oxygen supplementation. He received IV piperacillin-tazobactam for two weeks, with extended anti-TB therapy and continuous oral sulfamethoxazole and trimethoprim. As his symptoms persisted despite extensive antibiotic and anti-TB coverage, tests for cryptococcal antigen, prolonged culture for up to three weeks for *Nocardia* *spp*., and CMV DNA PCR were sent. Results were negative for Cryptococcal antigen and *Nocardia spp*.

**Figure 2:**
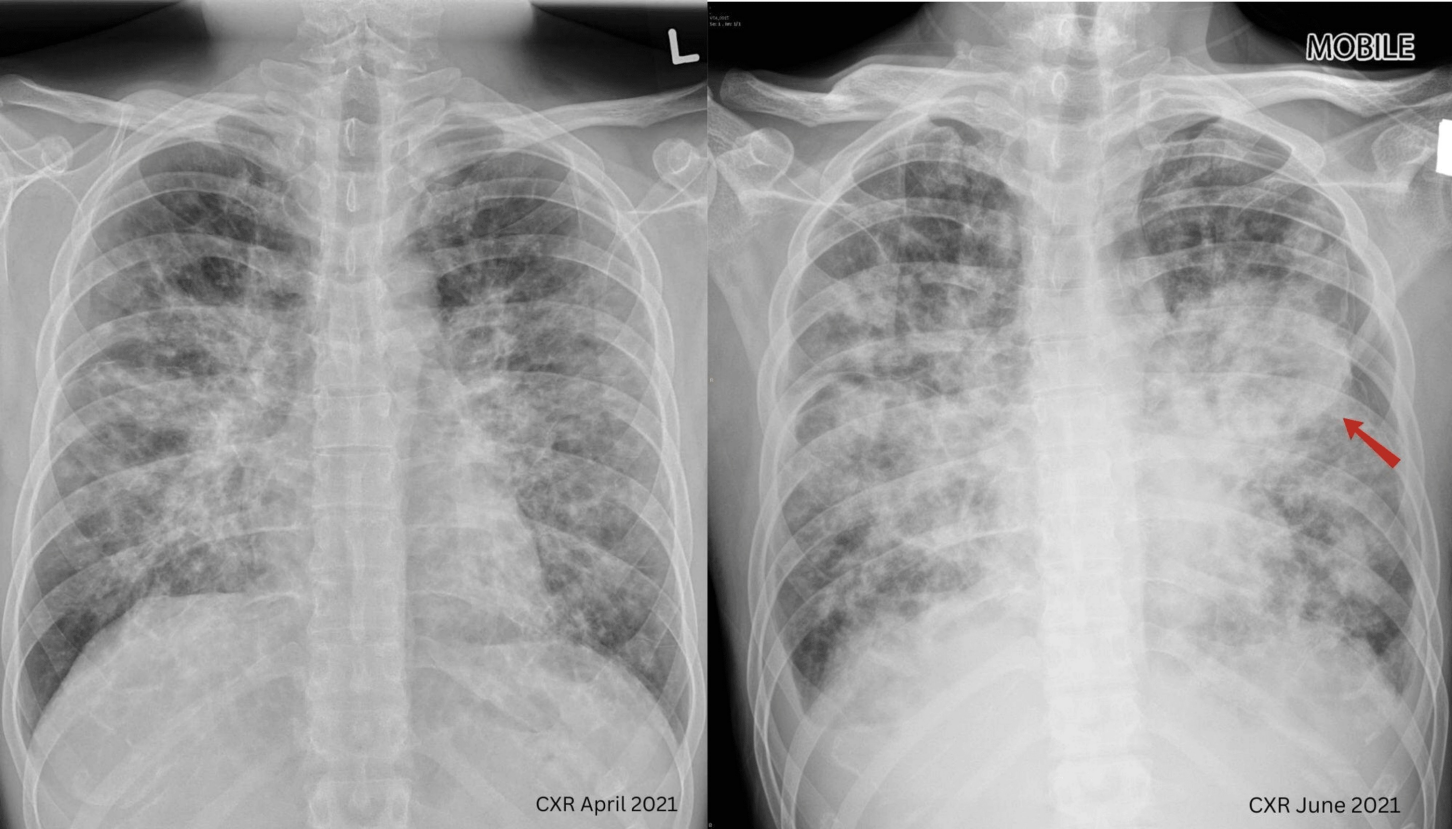
A comparison of the CXRs of the patient: first admission in April 2021 vs. June 2021 It demonstrates the progression of bilateral infiltrates from interstitial and nodular appearances to a mass-like infiltrate over the left side (red arrow). There was no radiological improvement from admission despite treatment with antibiotics, anti-TB, and treatment for PCP CXR: chest X-ray; PCP: pneumocystis pneumonia

**Figure 3:**
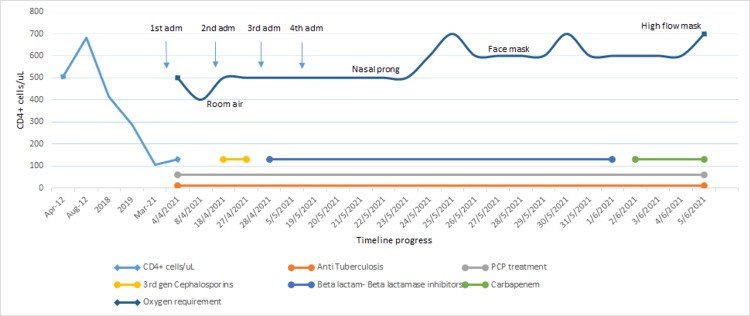
Timeline of patient’s progress throughout admission till his demise On the left side of the graph, the light-blue line illustrates the level of CD4+ in the patient from initial diagnosis of HIV in 2012 till admission to the hospital in 2021. On the upper part of the graph, the dark-blue line illustrates the progression of oxygen requirement throughout the multiple admissions to the hospital. The lower part of the graph shows the various treatments that were initiated from admission till his demise

A CXR on June 5, 2021, showed worsening consolidation with a circular opacity in the left lung (Figure 2). Amid a hospital COVID-19 outbreak, another COVID-19 PCR test was negative. A diagnostic bronchoscopy was not performed due to increasing oxygen requirements and COVID-19 restrictions. Antibiotics were escalated to IV meropenem, vancomycin, and azithromycin to cover for* Rhodococcus spp.* infection. Despite aggressive treatment, the patient’s condition deteriorated, leading to respiratory failure. Given his poor prognosis, a decision was made by consulting with the family to initiate palliative care. He ultimately passed away. His CMV DNA PCR returned low positive (<165 IU/mL, log 2.22). The patient’s clinical journey from admission to demise is summarized in Figure 3.

## Discussion

Despite the decreasing incidence of CMV infection in patients with AIDS, CMV remains a significant pathogen due to its interaction with HIV and its systemic immunomodulatory effects. Consequently, maintaining a high level of clinical vigilance for CMV infection is crucial [3]. Respiratory symptoms are common among HIV-infected individuals and are often caused by opportunistic infections. While typical pathogens such as bacterial infections, PCP, and TB are often considered, CMV-induced pneumonitis should also be high on the differential diagnosis. CMV pneumonitis typically presents as a subacute respiratory illness with non-specific symptoms such as fever, cough, dyspnea, and pulmonary infiltrates. It can also cause alveolar hemorrhage and more commonly, extrapulmonary manifestations, including retinitis, colitis, and hepatitis [4].

During our patient's third admission, he presented with symptoms consistent with colitis, which presumably could have been due to CMV, given the negative stool cultures for common pathogens. However, a delay in performing a CT scan of the abdomen and the inability to perform a colonoscopy for a tissue biopsy prevented a definitive diagnosis of CMV colitis due to COVID-19 limitations. An ophthalmological assessment also did not reveal CMV retinitis, which is often a common presentation of active CMV infection along with colitis. This lack of evidence for more common organ involvement further complicated the consensus on diagnosing CMV pneumonitis.

In patients with AIDS and CD4+ counts of <100 cells/μL, the presence of pulmonary infiltrates with an undetermined etiology or those progressing despite treatment for other identified pathogens should prompt consideration of probable CMV pneumonia, especially if CMV infection is documented at another site. Studies have shown that the lower the CD4+ count, the higher the prevalence of CMV, particularly in patients with CD4+ counts <50 cells/μL [5]. Our patient's latest CD4+ count was 132 cells/μL in April 2021, which initially made CMV a less likely cause of lung consolidation.

The histopathological features of pulmonary CMV infection differ between AIDS patients and immunocompromised transplant recipients. However, both groups typically present radiologically with bilateral infiltrates, which may be interstitial, nodular, or alveolar, often with a lower lobe predominance. This correlates with our patient's CTPA findings. Although the absence of small intrapulmonary cysts and apical distribution ruled out the likelihood of PCP infection [6], the mass-like consolidation observed on CXR was consistent with findings noted by McGuinness et al., who reported that mass-like infiltrates were more frequent in AIDS patients with CMV pneumonia.

A specific workup for CMV pneumonia is warranted if a patient with AIDS does not respond to empirical treatment for bacterial and PCP infections. Diagnosing CMV pneumonia accurately requires histological evidence of enlarged pulmonary cells with inclusion bodies, typically obtained via transbronchial biopsy [7]. However, bronchoalveolar lavage (BAL) cultures have low sensitivity and specificity for CMV diagnosis [8]. In terms of specificity, a positive CMV culture of BAL does not correlate with gas exchange, chest radiographic abnormalities, or acute morbidity due to pulmonary disease. Over half of BAL samples from HIV patients with pulmonary symptoms will contain CMV. Still, these patients do not have a more severe clinical presentation, hypoxemia, or three-week mortality compared to other forms of pneumonia.

In this case, we faced the challenge of diagnosing probable CMV pneumonia during the COVID-19 pandemic, which restricted the availability of aerosol-generating procedures such as bronchoscopy. The patient was also too ill to undergo the procedure, preventing us from obtaining a BAL specimen for a definitive diagnosis. This situation underscores the utility of quantitative PCR for CMV DNA, which provides a better estimate of viral burden and correlates with CD4+ levels and clinical symptoms [9]. Having said that, the serum CMV DNA PCR for this patient was sent late in the course of the disease and we only obtained the results a week after the patient's demise, which was <165 IU/mL (log 2.22).

Generally, the variability of the viral load ranges from 2log10 to 7log10 and anything less than 2log10 is considered “low positive”. However, clinical significance is difficult to interpret with low levels as they do not always correlate with disease development [10]. Now, reassessing the case retrospectively, even if the DNA PCR had been sent early, it would have added to the complexity of the diagnostic dilemma in our patient. Low viremia may translate into reactivation in a seriously ill patient, which does not always translate into organ disease, and therefore this could be probable CMV pneumonia.

Timely initiation of antiviral treatment, such as intravenous ganciclovir, should be strongly considered in patients with high clinical suspicion of CMV pneumonia, especially when invasive diagnostic procedures are not feasible. Empirical treatment can be life-saving, as delays in addressing CMV infection can lead to rapid deterioration and high mortality rates [11]. This approach is particularly critical in patients with low CD4+ counts and high HIV viral loads, who are at greater risk for severe CMV-related complications. Unfortunately, in our patient, the lack of definitive histopathological confirmation, combined with the inability to perform invasive procedures due to his critical condition and the COVID-19 pandemic restrictions, led to a delay in the diagnosis and treatment of CMV pneumonia. Consequently, the patient did not receive timely antiviral treatment and succumbed to the disease, underscoring the dire need for early empirical treatment in similar cases.

## Conclusions

This case highlights the complexity and uncertainties of diagnosing probable CMV pneumonia in AIDS and the various factors that hindered initiating early anti-CMV therapy during the era of the COVID-19 pandemic. The suspicion of probable CMV pneumonitis should be high in the context of AIDS and it should be carefully evaluated in patients with non-resolving pneumonia despite receiving adequate treatment for common pulmonary infections. Relevant radiological and cytohistopathological features should be worked out earlier in the disease course when the patient is stable. Empirical treatment should be considered early in ill patients while awaiting diagnostic tests.

## References

[1] Poh KC, Zheng S (2019). A rare case of CMV pneumonia in HIV-infection. Respir Med Case Rep.

[2] Zhao M, Zhuo C, Li Q, Liu L (2020). Cytomegalovirus (CMV) infection in HIV/AIDS patients and diagnostic values of CMV-DNA detection across different sample types. Ann Palliat Med.

[3] Tang HJ, Yu WL (2017). Cytomegalovirus pneumonia rather than pneumocystis pneumonia in a patient with human immunodeficiency virus infection: contributor or bystander?. J Infect Dis Epidemiol.

[4] Rodriguez-Barradas MC, Stool E, Musher DM, Gathe J Jr, Goldstein J, Genta RM, Yoffe B (1996). Diagnosing and treating cytomegalovirus pneumonia in patients with AIDS. Clin Infect Dis.

[5] Squire SB, Lipman MC, Bagdades EK, Mulvenna PM, Grundy JE, Griffiths PD, Johnson MA (1992). Severe cytomegalovirus pneumonitis in HIV infected patients with higher than average CD4 counts. Thorax.

[6] Deayton JR, Prof Sabin CA, Johnson MA, Emery VC, Wilson P, Griffiths PD (2004). Importance of cytomegalovirus viraemia in risk of disease progression and death in HIV-infected patients receiving highly active antiretroviral therapy. Lancet.

[7] Vogel MN, Brodoefel H, Hierl T, Beck R, Bethge WA, Claussen CD, Horger MS (2007). Differences and similarities of cytomegalovirus and pneumocystis pneumonia in HIV-negative immunocompromised patients thin section CT morphology in the early phase of the disease. Br J Radiol.

[8] Tan SK, Burgener EB, Waggoner JJ, Gajurel K, Gonzalez S, Chen SF, Pinsky BA (2016). Molecular and culture-based bronchoalveolar lavage fluid testing for the diagnosis of cytomegalovirus pneumonitis. Open Forum Infect Dis.

[9] Moon JH, Kim EA, Lee KS, Kim TS, Jung KJ, Song JH (2000). Cytomegalovirus pneumonia: high-resolution CT findings in ten non-AIDS immunocompromised patients. Korean J Radiol.

[10] Salomon N, Gomez T, Perlman DC, Laya L, Eber C, Mildvan D (1997). Clinical features and outcomes of HIV-related cytomegalovirus pneumonia. AIDS.

[11] McGuinness G, Scholes JV, Garay SM, Leitman BS, McCauley DI, Naidich DP (1994). Cytomegalovirus pneumonitis: spectrum of parenchymal CT findings with pathologic correlation in 21 AIDS patients. Radiology.

